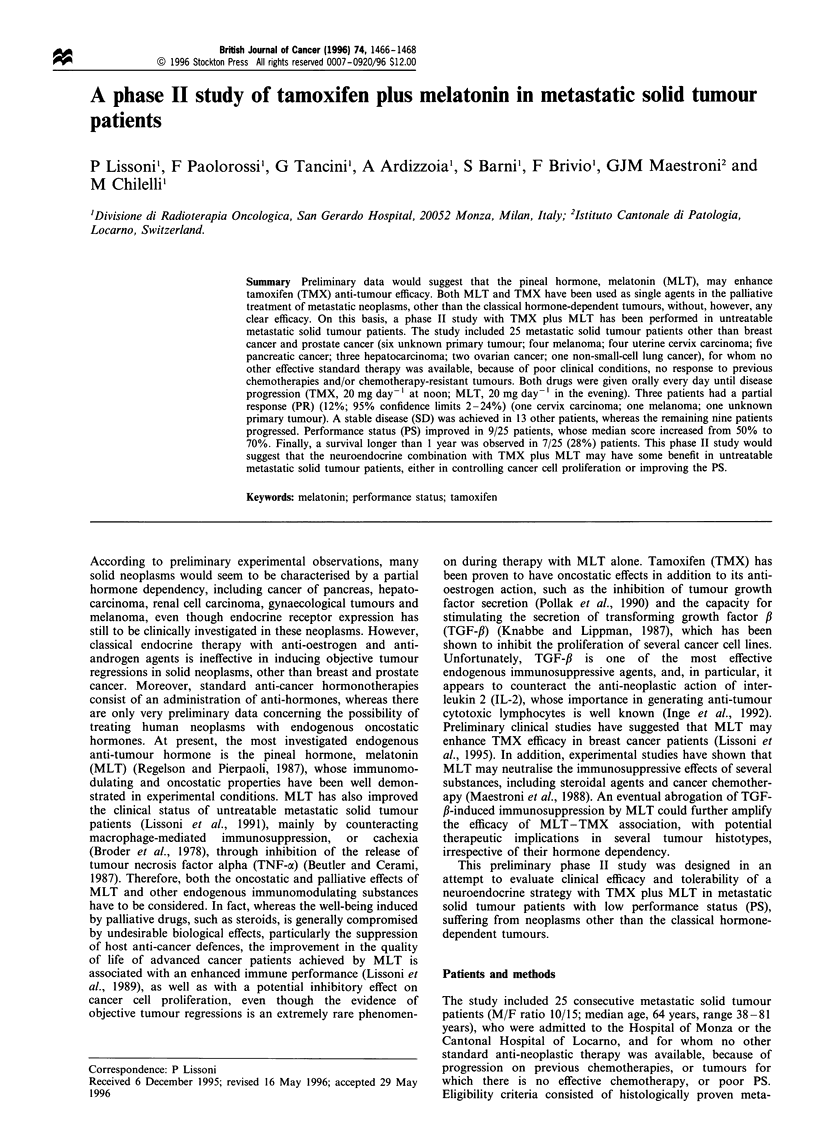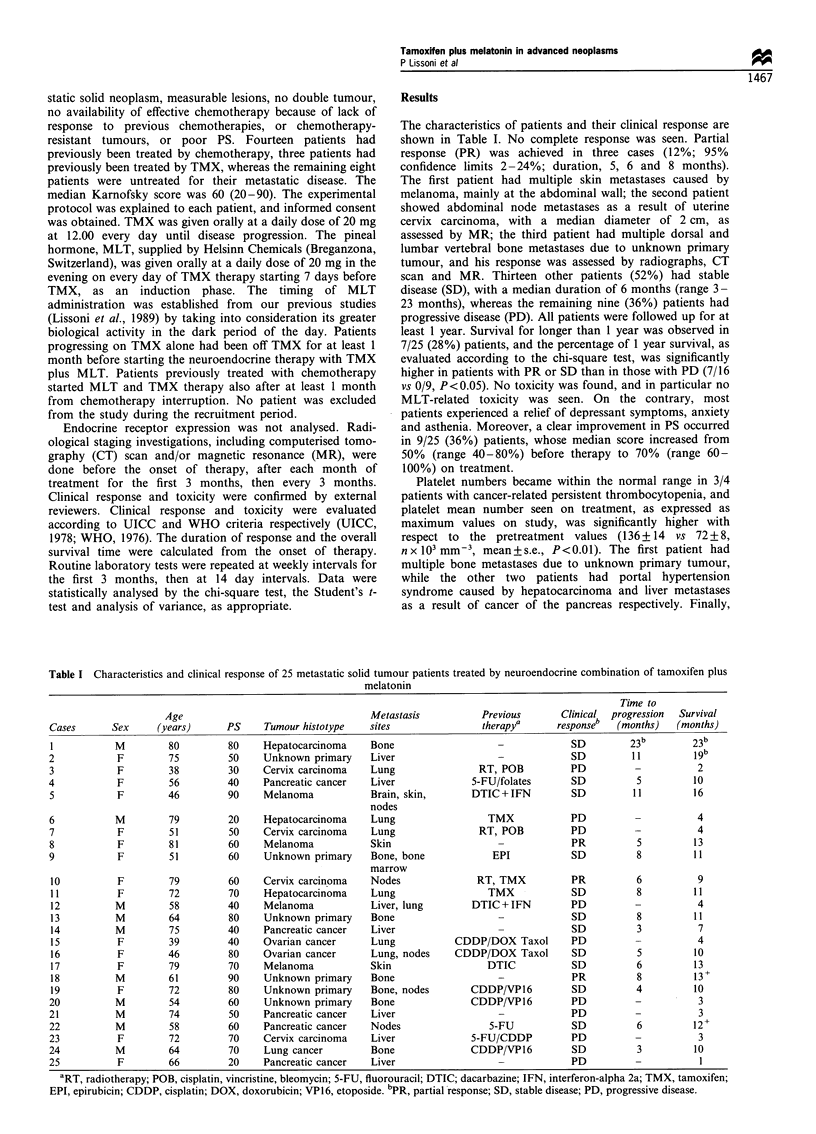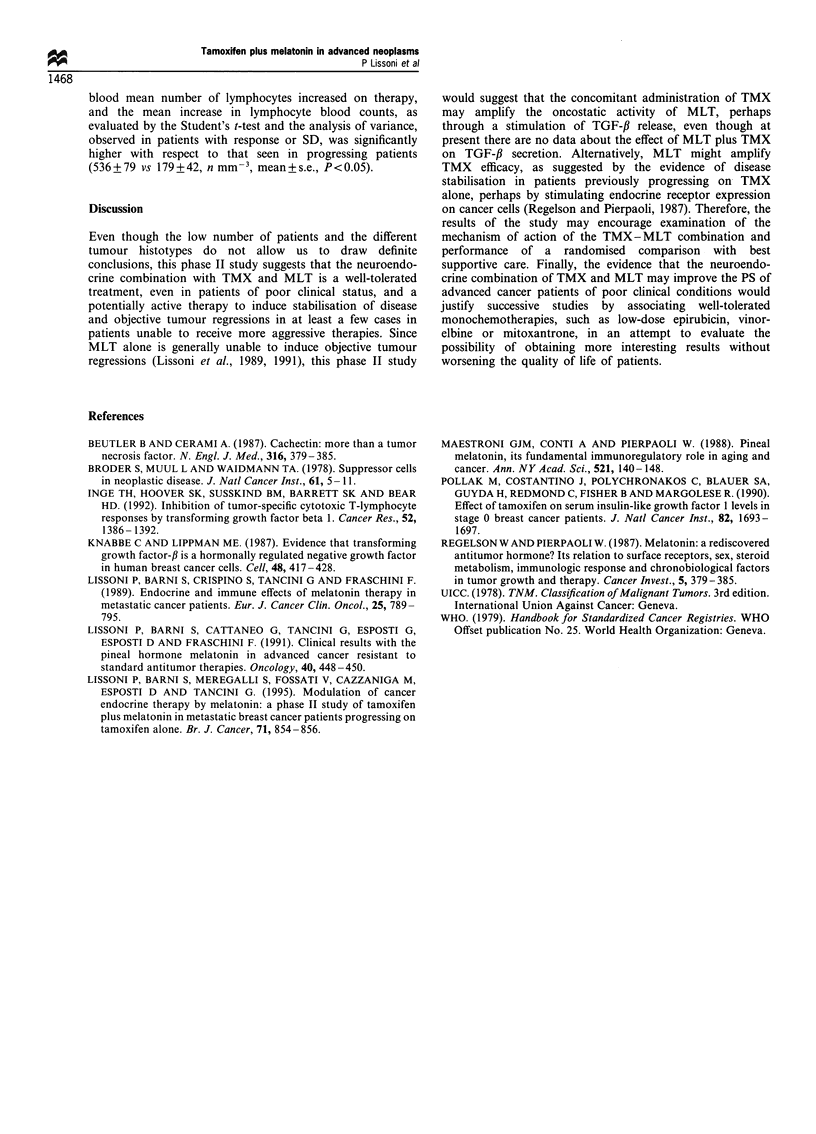# A phase II study of tamoxifen plus melatonin in metastatic solid tumour patients.

**DOI:** 10.1038/bjc.1996.566

**Published:** 1996-11

**Authors:** P. Lissoni, F. Paolorossi, G. Tancini, A. Ardizzoia, S. Barni, F. Brivio, G. J. Maestroni, M. Chilelli

**Affiliations:** Divisione di Radioterapia Oncologica, San Gerardo Hospital, Monza, Milan, Italy.

## Abstract

Preliminary data would suggest that the pineal hormone, melatonin (MLT), may enhance tamoxifen (TMX) anti-tumour efficacy. Both MLT and TMX have been used as single agents in the palliative treatment of metastatic neoplasms, other than the classical hormone-dependent tumours, without, however, any clear efficacy. On this basis, a phase II study with TMX plus MLT has been performed in untreatable metastatic solid tumour patients. The study included 25 metastatic solid tumour patients other than breast cancer and prostate cancer (six unknown primary tumour; four melanoma; four uterine cervix carcinoma; five pancreatic cancer; three hepatocarcinoma; two ovarian cancer; one non-small-cell lung cancer), for whom no other effective standard therapy was available, because of poor clinical conditions, no response to previous chemotherapies and/or chemotherapy-resistant tumours. Both drugs were given orally every day until disease progression (TMX, 20 mg day-1 at noon; MLT, 20 mg day-1 in the evening). Three patients had a partial response (PR) (12%; 95% confidence limits 2-24%) (one cervix carcinoma; one melanoma; one unknown primary tumour). A stable disease (SD) was achieved in 13 other patients, whereas the remaining nine patients progressed. Performance status (PS) improved in 9/25 patients, whose median score increased from 50% to 70%. Finally, a survival longer than 1 year was observed in 7/25 (28%) patients. This phase II study would suggest that the neuroendocrine combination with TMX plus MLT may have some benefit in untreatable metastatic solid tumour patients, either in controlling cancer cell proliferation or improving the PS.


British Journal of Cancer (1996) 74, 1466-1468
© 1996 Stockton Press All rights reserved 0007-0920/96 $12.00

### A phase II study of tamoxifen plus melatonin in metastatic solid tumour patients

P Lissoni[1], F Paolorossi[1], G Tancini[1], A Ardizzoia[1], S Barni[1], F Brivio[1], GJM Maestroni[2] and M Chilelli[1]

[1]*Divisione di Radioterapia Oncologica, San Gerardo Hospital, 20052 Monza, Milan, Italy;* [2]*Istituto Cantonale di Patologia, Locarno, Switzerland.*

**Summary** Preliminary data would suggest that the pineal hormone, melatonin (MLT), may enhance tamoxifen (TMX) anti-tumour efficacy. Both MLT and TMX have been used as single agents in the palliative treatment of metastatic neoplasms, other than the classical hormone-dependent tumours, without, however, any clear efficacy. On this basis, a phase II study with TMX plus MLT has been performed in untreatable metastatic solid tumour patients. The study included 25 metastatic solid tumour patients other than breast cancer and prostate cancer (six unknown primary tumour; four melanoma; four uterine cervix carcinoma; five pancreatic cancer; three hepatocarcinoma; two ovarian cancer; one non-small-cell lung cancer), for whom no other effective standard therapy was available, because of poor clinical conditions, no response to previous chemotherapies and/or chemotherapy-resistant tumours. Both drugs were given orally every day until disease progression (TMX, 20 mg day$^{-1}$ at noon; MLT, 20 mg day$^{-1}$ in the evening). Three patients had a partial response (PR) (12%; 95% confidence limits 2–24%) (one cervix carcinoma; one melanoma; one unknown primary tumour). A stable disease (SD) was achieved in 13 other patients, whereas the remaining nine patients progressed. Performance status (PS) improved in 9/25 patients, whose median score increased from 50% to 70%. Finally, a survival longer than 1 year was observed in 7/25 (28%) patients. This phase II study would suggest that the neuroendocrine combination with TMX plus MLT may have some benefit in untreatable metastatic solid tumour patients, either in controlling cancer cell proliferation or improving the PS.

**Keywords:** melatonin; performance status; tamoxifen

According to preliminary experimental observations, many solid neoplasms would seem to be characterised by a partial hormone dependency, including cancer of pancreas, hepatocarcinoma, renal cell carcinoma, gynaecological tumours and melanoma, even though endocrine receptor expression has still to be clinically investigated in these neoplasms. However, classical endocrine therapy with anti-oestrogen and anti-androgen agents is ineffective in inducing objective tumour regressions in solid neoplasms, other than breast and prostate cancer. Moreover, standard anti-cancer hormonotherapies consist of an administration of anti-hormones, whereas there are only very preliminary data concerning the possibility of treating human neoplasms with endogenous oncostatic hormones. At present, the most investigated endogenous anti-tumour hormone is the pineal hormone, melatonin (MLT) (Regelson and Pierpaoli, 1987), whose immunomodulating and oncostatic properties have been well demonstrated in experimental conditions. MLT has also improved the clinical status of untreatable metastatic solid tumour patients (Lissoni *et al.*, 1991), mainly by counteracting macrophage-mediated immunosuppression, or cachexia (Broder *et al.*, 1978), through inhibition of the release of tumour necrosis factor alpha (TNF-$\alpha$) (Beutler and Cerami, 1987). Therefore, both the oncostatic and palliative effects of MLT and other endogenous immunomodulating substances have to be considered. In fact, whereas the well-being induced by palliative drugs, such as steroids, is generally compromised by undesirable biological effects, particularly the suppression of host anti-cancer defences, the improvement in the quality of life of advanced cancer patients achieved by MLT is associated with an enhanced immune performance (Lissoni *et al.*, 1989), as well as with a potential inhibitory effect on cancer cell proliferation, even though the evidence of objective tumour regressions is an extremely rare phenomenon during therapy with MLT alone. Tamoxifen (TMX) has been proven to have oncostatic effects in addition to its anti-oestrogen action, such as the inhibition of tumour growth factor secretion (Pollak *et al.*, 1990) and the capacity for stimulating the secretion of transforming growth factor $\beta$ (TGF-$\beta$) (Knabbe and Lippman, 1987), which has been shown to inhibit the proliferation of several cancer cell lines. Unfortunately, TGF-$\beta$ is one of the most effective endogenous immunosuppressive agents, and, in particular, it appears to counteract the anti-neoplastic action of interleukin 2 (IL-2), whose importance in generating anti-tumour cytotoxic lymphocytes is well known (Inge *et al.*, 1992). Preliminary clinical studies have suggested that MLT may enhance TMX efficacy in breast cancer patients (Lissoni *et al.*, 1995). In addition, experimental studies have shown that MLT may neutralise the immunosuppressive effects of several substances, including steroidal agents and cancer chemotherapy (Maestroni *et al.*, 1988). An eventual abrogation of TGF-$\beta$-induced immunosuppression by MLT could further amplify the efficacy of MLT–TMX association, with potential therapeutic implications in several tumour histotypes, irrespective of their hormone dependency.

This preliminary phase II study was designed in an attempt to evaluate clinical efficacy and tolerability of a neuroendocrine strategy with TMX plus MLT in metastatic solid tumour patients with low performance status (PS), suffering from neoplasms other than the classical hormone-dependent tumours.

### Patients and methods

The study included 25 consecutive metastatic solid tumour patients (M/F ratio 10/15; median age, 64 years, range 38–81 years), who were admitted to the Hospital of Monza or the Cantonal Hospital of Locarno, and for whom no other standard anti-neoplastic therapy was available, because of progression on previous chemotherapies, or tumours for which there is no effective chemotherapy, or poor PS. Eligibility criteria consisted of histologically proven meta-

Correspondence: P Lissoni
Received 6 December 1995; revised 16 May 1996; accepted 29 May 1996

static solid neoplasm, measurable lesions, no double tumour, no availability of effective chemotherapy because of lack of response to previous chemotherapies, or chemotherapy-resistant tumours, or poor PS. Fourteen patients had previously been treated by chemotherapy, three patients had previously been treated by TMX, whereas the remaining eight patients were untreated for their metastatic disease. The median Karnofsky score was 60 (20–90). The experimental protocol was explained to each patient, and informed consent was obtained. TMX was given orally at a daily dose of 20 mg at 12.00 every day until disease progression. The pineal hormone, MLT, supplied by Helsinn Chemicals (Breganzona, Switzerland), was given orally at a daily dose of 20 mg in the evening on every day of TMX therapy starting 7 days before TMX, as an induction phase. The timing of MLT administration was established from our previous studies (Lissoni *et al*., 1989) by taking into consideration its greater biological activity in the dark period of the day. Patients progressing on TMX alone had been off TMX for at least 1 month before starting the neuroendocrine therapy with TMX plus MLT. Patients previously treated with chemotherapy started MLT and TMX therapy also after at least 1 month from chemotherapy interruption. No patient was excluded from the study during the recruitment period.

Endocrine receptor expression was not analysed. Radiological staging investigations, including computerised tomography (CT) scan and/or magnetic resonance (MR), were done before the onset of therapy, after each month of treatment for the first 3 months, then every 3 months. Clinical response and toxicity were confirmed by external reviewers. Clinical response and toxicity were evaluated according to UICC and WHO criteria respectively (UICC, 1978; WHO, 1976). The duration of response and the overall survival time were calculated from the onset of therapy. Routine laboratory tests were repeated at weekly intervals for the first 3 months, then at 14 day intervals. Data were statistically analysed by the chi-square test, the Student's *t*-test and analysis of variance, as appropriate.

### Results

The characteristics of patients and their clinical response are shown in Table I. No complete response was seen. Partial response (PR) was achieved in three cases (12%; 95% confidence limits 2–24%; duration, 5, 6 and 8 months). The first patient had multiple skin metastases caused by melanoma, mainly at the abdominal wall; the second patient showed abdominal node metastases as a result of uterine cervix carcinoma, with a median diameter of 2 cm, as assessed by MR; the third patient had multiple dorsal and lumbar vertebral bone metastases due to unknown primary tumour, and his response was assessed by radiographs, CT scan and MR. Thirteen other patients (52%) had stable disease (SD), with a median duration of 6 months (range 3–23 months), whereas the remaining nine (36%) patients had progressive disease (PD). All patients were followed up for at least 1 year. Survival for longer than 1 year was observed in 7/25 (28%) patients, and the percentage of 1 year survival, as evaluated according to the chi-square test, was significantly higher in patients with PR or SD than in those with PD (7/16 *vs* 0/9, $P<0.05$). No toxicity was found, and in particular no MLT-related toxicity was seen. On the contrary, most patients experienced a relief of depressant symptoms, anxiety and asthenia. Moreover, a clear improvement in PS occurred in 9/25 (36%) patients, whose median score increased from 50% (range 40–80%) before therapy to 70% (range 60–100%) on treatment.

Platelet numbers became within the normal range in 3/4 patients with cancer-related persistent thrombocytopenia, and platelet mean number seen on treatment, as expressed as maximum values on study, was significantly higher with respect to the pretreatment values ($136\pm14$ *vs* $72\pm8$, $n\times10^3$ $mm^{-3}$, mean$\pm$s.e., $P<0.01$). The first patient had multiple bone metastases due to unknown primary tumour, while the other two patients had portal hypertension syndrome caused by hepatocarcinoma and liver metastases as a result of cancer of the pancreas respectively. Finally,

**Table I** Characteristics and clinical response of 25 metastatic solid tumour patients treated by neuroendocrine combination of tamoxifen plus melatonin

| *Cases* | *Sex* | *Age (years)* | *PS* | *Tumour histotype* | *Metastasis sites* | *Previous therapy*[a] | *Clinical response*[b] | *Time to progression (months)* | *Survival (months)* |
|---|---|---|---|---|---|---|---|---|---|
| 1 | M | 80 | 80 | Hepatocarcinoma | Bone | – | SD | 23[b] | 23[b] |
| 2 | F | 75 | 50 | Unknown primary | Liver | – | SD | 11 | 19[b] |
| 3 | F | 38 | 30 | Cervix carcinoma | Lung | RT, POB | PD | – | 2 |
| 4 | F | 56 | 40 | Pancreatic cancer | Liver | 5-FU/folates | SD | 5 | 10 |
| 5 | F | 46 | 90 | Melanoma | Brain, skin, nodes | DTIC+IFN | SD | 11 | 16 |
| 6 | M | 79 | 20 | Hepatocarcinoma | Lung | TMX | PD | – | 4 |
| 7 | F | 51 | 50 | Cervix carcinoma | Lung | RT, POB | PD | – | 4 |
| 8 | F | 81 | 60 | Melanoma | Skin | – | PR | 5 | 13 |
| 9 | F | 51 | 60 | Unknown primary | Bone, bone marrow | EPI | SD | 8 | 11 |
| 10 | F | 79 | 60 | Cervix carcinoma | Nodes | RT, TMX | PR | 6 | 9 |
| 11 | F | 72 | 70 | Hepatocarcinoma | Lung | TMX | SD | 8 | 11 |
| 12 | M | 58 | 40 | Melanoma | Liver, lung | DTIC+IFN | PD | – | 4 |
| 13 | M | 64 | 80 | Unknown primary | Bone | – | SD | 8 | 11 |
| 14 | M | 75 | 40 | Pancreatic cancer | Liver | – | SD | 3 | 7 |
| 15 | F | 39 | 40 | Ovarian cancer | Lung | CDDP/DOX Taxol | PD | – | 4 |
| 16 | F | 46 | 80 | Ovarian cancer | Lung, nodes | CDDP/DOX Taxol | SD | 5 | 10 |
| 17 | F | 79 | 70 | Melanoma | Skin | DTIC | SD | 6 | 13 |
| 18 | M | 61 | 90 | Unknown primary | Bone | – | PR | 8 | 13[+] |
| 19 | F | 72 | 80 | Unknown primary | Bone, nodes | CDDP/VP16 | SD | 4 | 10 |
| 20 | M | 54 | 60 | Unknown primary | Bone | CDDP/VP16 | PD | – | 3 |
| 21 | M | 74 | 50 | Pancreatic cancer | Liver | – | PD | – | 3 |
| 22 | M | 58 | 60 | Pancreatic cancer | Nodes | 5-FU | SD | 6 | 12[+] |
| 23 | F | 72 | 70 | Cervix carcinoma | Liver | 5-FU/CDDP | PD | – | 3 |
| 24 | M | 64 | 70 | Lung cancer | Bone | CDDP/VP16 | SD | 3 | 10 |
| 25 | F | 66 | 20 | Pancreatic cancer | Liver | – | PD | – | 1 |

[a]RT, radiotherapy; POB, cisplatin, vincristine, bleomycin; 5-FU, fluorouracil; DTIC; dacarbazine; IFN, interferon-alpha 2a; TMX, tamoxifen; EPI, epirubicin; CDDP, cisplatin; DOX, doxorubicin; VP16, etoposide. [b]PR, partial response; SD, stable disease; PD, progressive disease.

blood mean number of lymphocytes increased on therapy, and the mean increase in lymphocyte blood counts, as evaluated by the Student's $t$-test and the analysis of variance, observed in patients with response or SD, was significantly higher with respect to that seen in progressing patients ($536 \pm 79$ *vs* $179 \pm 42$, $n$ $mm^{-3}$, mean$\pm$s.e., $P < 0.05$).

### Discussion

Even though the low number of patients and the different tumour histotypes do not allow us to draw definite conclusions, this phase II study suggests that the neuroendocrine combination with TMX and MLT is a well-tolerated treatment, even in patients of poor clinical status, and a potentially active therapy to induce stabilisation of disease and objective tumour regressions in at least a few cases in patients unable to receive more aggressive therapies. Since MLT alone is generally unable to induce objective tumour regressions (Lissoni *et al.*, 1989, 1991), this phase II study would suggest that the concomitant administration of TMX may amplify the oncostatic activity of MLT, perhaps through a stimulation of TGF-$\beta$ release, even though at present there are no data about the effect of MLT plus TMX on TGF-$\beta$ secretion. Alternatively, MLT might amplify TMX efficacy, as suggested by the evidence of disease stabilisation in patients previously progressing on TMX alone, perhaps by stimulating endocrine receptor expression on cancer cells (Regelson and Pierpaoli, 1987). Therefore, the results of the study may encourage examination of the mechanism of action of the TMX–MLT combination and performance of a randomised comparison with best supportive care. Finally, the evidence that the neuroendocrine combination of TMX and MLT may improve the PS of advanced cancer patients of poor clinical conditions would justify successive studies by associating well-tolerated monochemotherapies, such as low-dose epirubicin, vinorelbine or mitoxantrone, in an attempt to evaluate the possibility of obtaining more interesting results without worsening the quality of life of patients.

### References

BEUTLER B AND CERAMI A. (1987). Cachectin: more than a tumor necrosis factor. *N. Engl. J. Med.*, **316,** 379–385.

BRODER S, MUUL L AND WAIDMANN TA. (1978). Suppressor cells in neoplastic disease. *J. Natl Cancer Inst.*, **61,** 5–11.

INGE TH, HOOVER SK, SUSSKIND BM, BARRETT SK AND BEAR HD. (1992). Inhibition of tumor-specific cytotoxic T-lymphocyte responses by transforming growth factor beta 1. *Cancer Res.*, **52,** 1386–1392.

KNABBE C AND LIPPMAN ME. (1987). Evidence that transforming growth factor-$\beta$ is a hormonally regulated negative growth factor in human breast cancer cells. *Cell*, **48,** 417–428.

LISSONI P, BARNI S, CRISPINO S, TANCINI G AND FRASCHINI F. (1989). Endocrine and immune effects of melatonin therapy in metastatic cancer patients. *Eur. J. Cancer Clin. Oncol.*, **25,** 789–795.

LISSONI P, BARNI S, CATTANEO G, TANCINI G, ESPOSTI G, ESPOSTI D AND FRASCHINI F. (1991). Clinical results with the pineal hormone melatonin in advanced cancer resistant to standard antitumor therapies. *Oncology*, **40,** 448–450.

LISSONI P, BARNI S, MEREGALLI S, FOSSATI V, CAZZANIGA M, ESPOSTI D AND TANCINI G. (1995). Modulation of cancer endocrine therapy by melatonin: a phase II study of tamoxifen plus melatonin in metastatic breast cancer patients progressing on tamoxifen alone. *Br. J. Cancer*, **71,** 854–856.

MAESTRONI GJM, CONTI A AND PIERPAOLI W. (1988). Pineal melatonin, its fundamental immunoregulatory role in aging and cancer. *Ann. NY Acad. Sci.*, **521,** 140–148.

POLLAK M, COSTANTINO J, POLYCHRONAKOS C, BLAUER SA, GUYDA H, REDMOND C, FISHER B AND MARGOLESE R. (1990). Effect of tamoxifen on serum insulin-like growth factor 1 levels in stage 0 breast cancer patients. *J. Natl Cancer Inst.*, **82,** 1693–1697.

REGELSON W AND PIERPAOLI W. (1987). Melatonin: a rediscovered antitumor hormone? Its relation to surface receptors, sex, steroid metabolism, immunologic response and chronobiological factors in tumor growth and therapy. *Cancer Invest.*, **5,** 379–385.

UICC. (1978). *TNM. Classification of Malignant Tumors.* 3rd edition. International Union Against Cancer: Geneva.

WHO. (1979). *Handbook for Standardized Cancer Registries.* WHO Offset publication No. 25. World Health Organization: Geneva.